# Supplementary material for: Kukoamine B from Lycii Radicis Cortex Protects Human Keratinocyte HaCaT Cells through Covalent Modification by Trans-2-Nonenal
Source: Plants (Basel). 2022 Dec 29;12(1):163. doi: 10.3390/plants12010163 (PMC9823295; doi:10.3390/plants12010163)
Supplement: Supplementary file 1 [file plants-12-00163-s001.zip › plants-1999258-supplementary.pdf]

Supplementary Material

Kukoamine B from Lycii Radicis Cortex protects human keratinocytes HaCaT cells through covalent modification by *trans*-2-nonenal

Hye Mi Kim[1], Jae-Yong Kim[1], Ji Hoon Kim[1] and Chul Young Kim[1,*]

[1]College of Pharmacy and Institute of Pharmaceutical Science and Technology, Hanyang University, Ansan, Gyeonggi-do 15588, Republic of Korea

*Corresponding author : Chul Young Kim

E-mail address: chulykim@hanyang.ac.kr

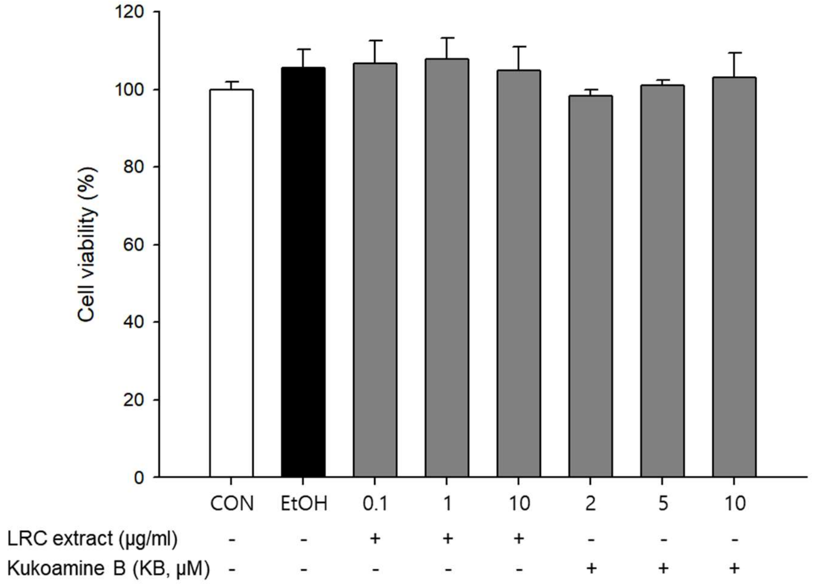

Figure S1. Cell viability of human keratinocytes treated with LRC extract (0.1, 1, 10 μg/ml) or KB (2, 5, 10 μM). These data are expressed as mean ± SD of three independent experiments.

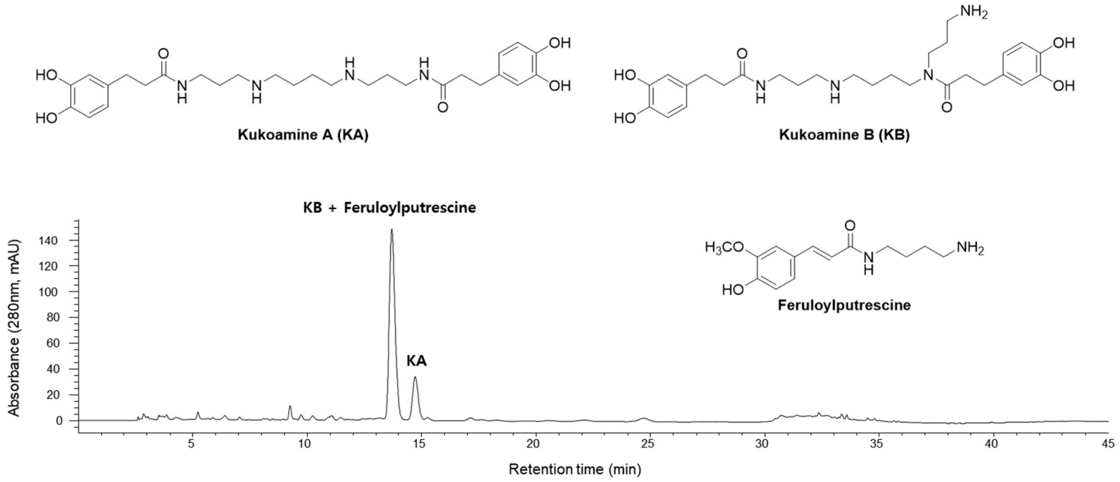

Figure S2. The HPLC chromatogram monitored at 280 nm of the LRC extract (10 mg/ml) dissolved in PBS.

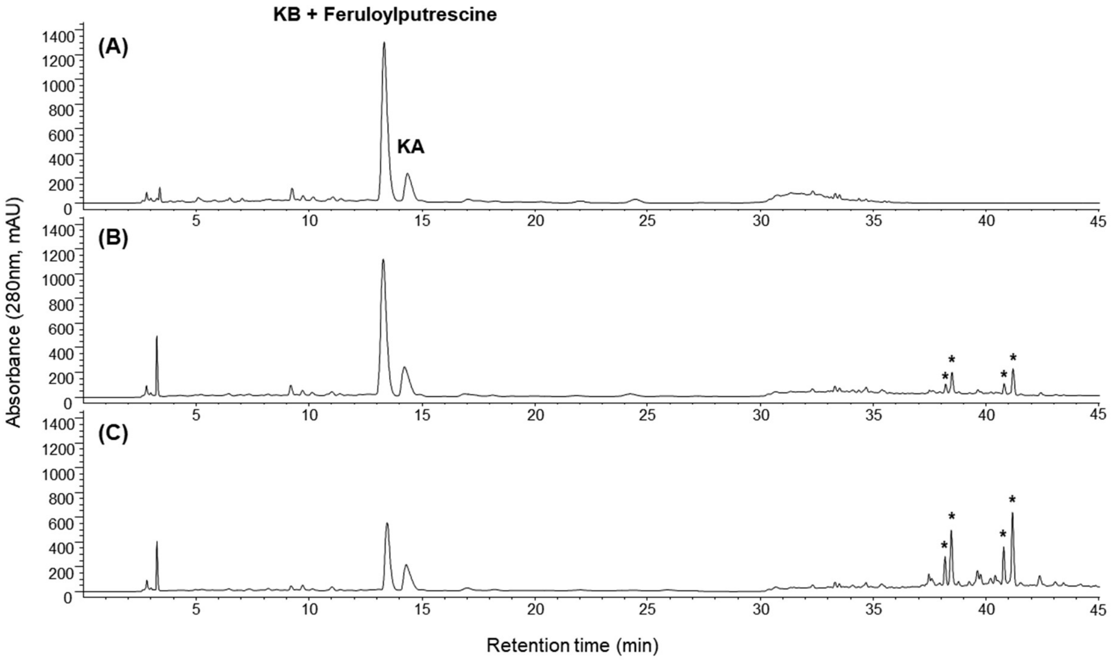

Figure S3. The HPLC chromatogram of LRC extract (100 mg/ml) after incubation with (**A**) 0.1 mM, (**B**) 50 mM, and (**C**) 100 mM of *trans*-2-nonenal for 24 h. The peaks designated as asterisk (*) were new product peaks appeared though this reaction.

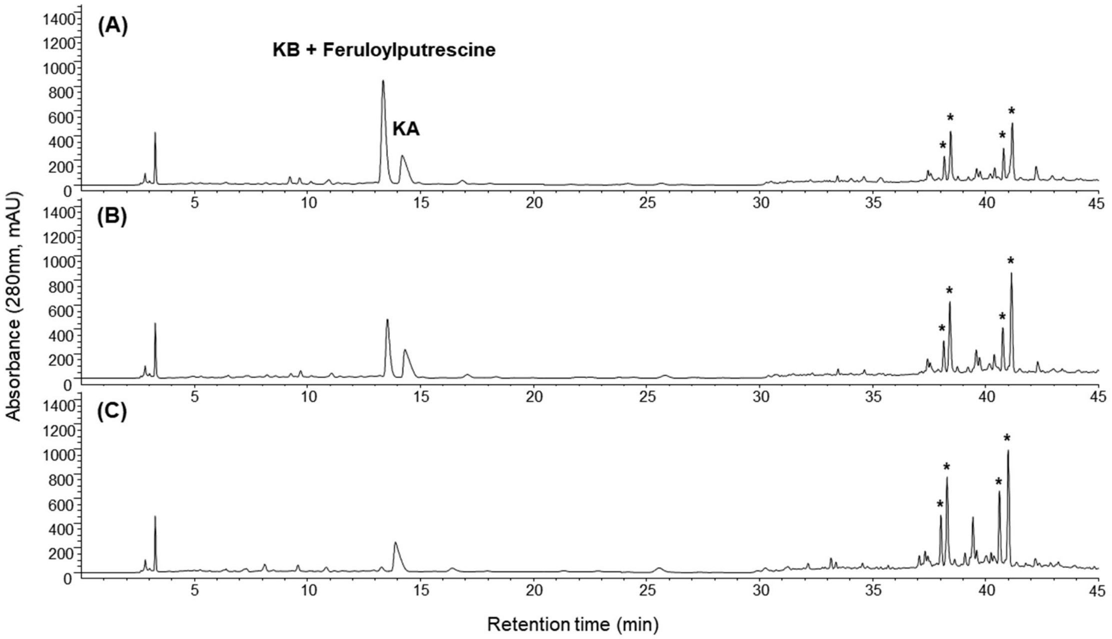

Figure S4. The HPLC chromatogram of LRC extract (100 mg/ml) after incubation with 100 mM of *trans*-2-nonenal for (**A**) 24 h, (**B**) 48 h, and (**C**) 72 h, respectively. The peaks designated as asterisk (*) were new product peaks appeared though this reaction.

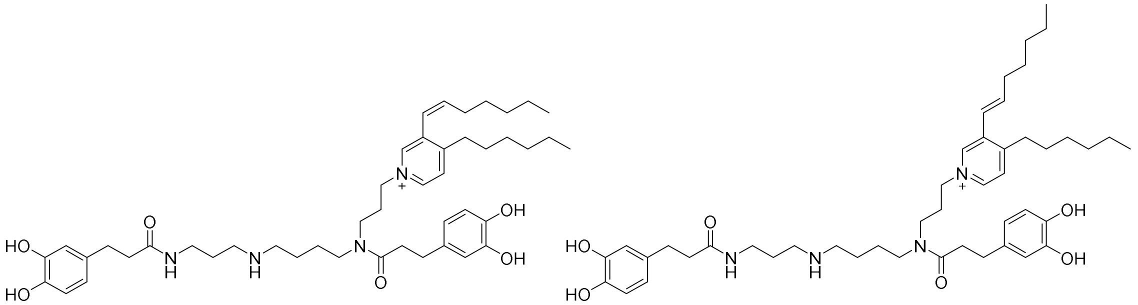

***cis*-Adduct 1** (*m/z* 773.5) ***trans*-Adduct 1** (*m/w* 773.5)

Figure S5. Structures of the possible KB-modified adducts by *trans*-2-nonenal.

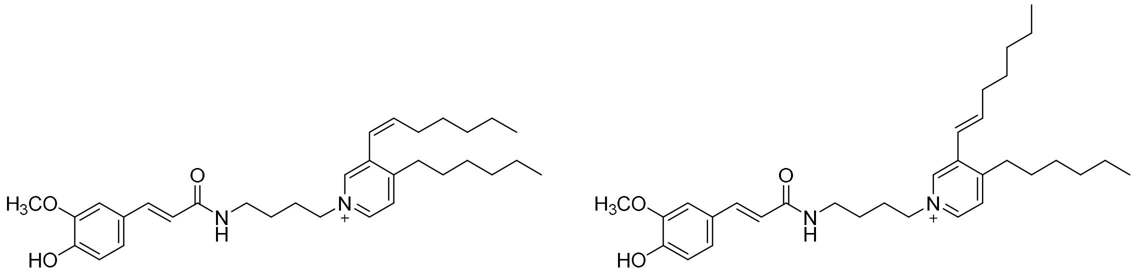

***cis*-Adduct 2** (*m/z* 507.4) ***trans*-Adduct 2** (*m/z* 507.4)

Figure S6. Structures of the possible feruloylputrescine-modified adducts by *trans*-2-nonenal.

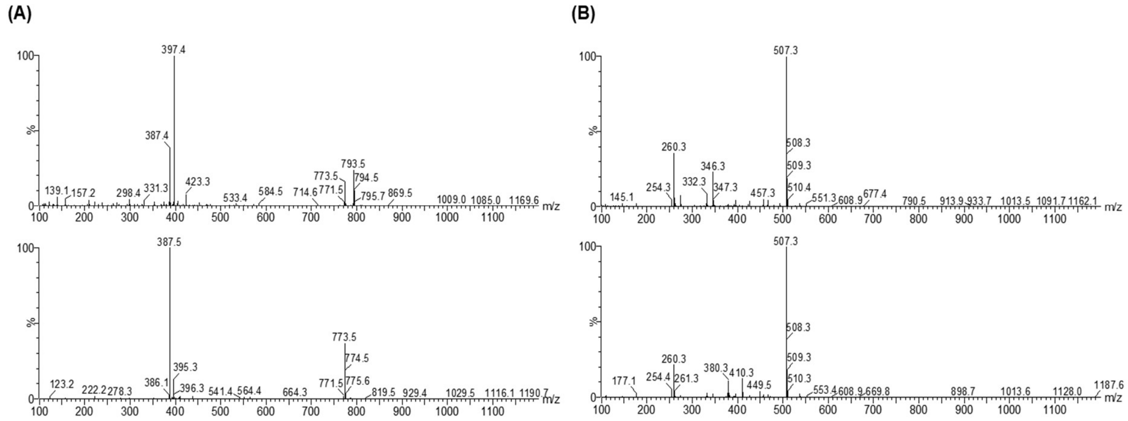

Figure S7. LC-ESI-MS spectra of the (**A**) KB and (**B**) feruloylputrescine-modified adducts by *trans*-2-nonenal.

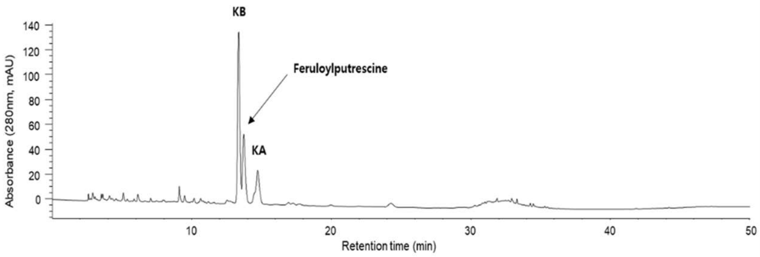

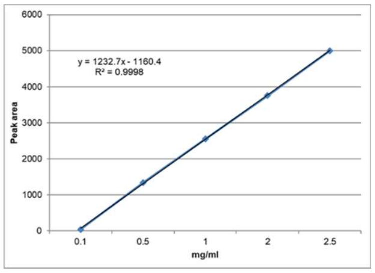

Figure S8. HPLC-UV profiles of the extracts and calibration curve of KB (R^2=0.9998).
